# GDP‐fucose transporter SLC35C1: a potential regulatory role in cytosolic GDP‐fucose and fucosylated glycan synthesis

**DOI:** 10.1002/2211-5463.70057

**Published:** 2025-05-27

**Authors:** Edyta Skurska, Mariusz Olczak

**Affiliations:** ^1^ Faculty of Biotechnology University of Wroclaw Poland

**Keywords:** carbohydrate metabolism, FCSK, fucosylation, GMDS, SLC35C1, TSTA3

## Abstract

Glycosylation occurs mainly in the Golgi apparatus, whereas the synthesis of nucleotide sugars occurs in the cytoplasm or nucleus. GDP‐fucose in mammalian cells could be produced via *de novo* and salvage pathways in the cytoplasm; the first one is responsible for about 90% of GDP‐fucose in the total pool of this nucleotide sugar in the cell. SLC35C1 (C1) is the primary transporter of GDP‐fucose to the Golgi apparatus. In the absence of this transporter, it was proposed that nucleotide sugar could still reach the Golgi apparatus via a SLC35C2, the homologue of SLC35C1. However, simultaneous inactivation of the two transporters did not influence GDP‐fucose transport across the Golgi apparatus membranes after external fucose supplementation. In this study, we combined the inactivation of SLC35C1 and enzymes of the GDP‐fucose biosynthesis pathways (FCSK, GMDS and TSTA3) to study the impact of double inactivation on the production of nucleotide sugar and fucosylated glycans. We found that a lack of SLC35C1 changed the level of enzymes of both *de novo* and salvage pathways. Upon fucose supplementation, stimulation of the salvage pathway was remarkably high in the absence of the TSTA3 protein, and the concentration of GDP‐fucose increased to millimolar values. In this work, we discovered that simultaneous deficiency of the SLC35C1 protein and TSTA3 enzyme increased GDP‐fucose production via the salvage pathway to an even higher level. Finally, we found that nucleotide sugar still accessed the Golgi apparatus and had differential effects on *N*‐ and *O*‐glycans.

## Graphical abstract

The inactivation of SLC35C1 (GDP‐fucose transporter) and enzymes involved in GDP‐fucose biosynthesis was studied. Fucose supplementation increases the level of GDP‐fucose to abnormal, millimolar values in the absence of the TSTA3 protein and SLC35C1 in contrast to the GMDS/SLC35C1 double mutant. GDP‐fucose still accesses the lumen of the Golgi apparatus but has differential effects on glycosylation when compared to wild‐type cells.

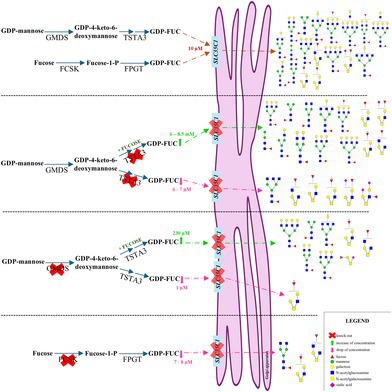

**Abbreviations**
2‐AB: 2‐aminobenzamide
ATCC: American Type Culture Collection
C1: SLC35C1 protein
Cas9: CRISPR‐associated protein 9 complementary DNA
CDG: congenital disorder of glycosylation
DMEM: Dulbecco's modified Eagle's medium
EMT: epithelial‐mesenchymal transition
FCSK: fucokinase
FPGT: fucose‐1‐phosphate guanylyltransferase
GALE: UDP‐galactose‐4‐epimerase
GALK: galactokinase
GALT: galactose‐1‐phosphate uridylyltransferase
GMDS: GDP‐mannose 4,6‐dehydratase
HEK293T: human embryonic kidney 293T cell line
HPLC: high‐performance liquid chromatograph
MALDI: matrix‐assisted laser desorption/ionisation
NS: nucleotide sugar
PBST: phosphate‐buffered saline with Tween 20
SLC35: solute carriers 35 family
TM: transmembrane
TOF: time of flight
TSTA3: GDP‐fucose synthase also known as FX protein, SDR4E1, p35B or GFUS
WT: wild type

Glycosylation is a common modification of macromolecules in mammalian cells. It is estimated that over half of all proteins are glycosylated, and 1–2% of the genome encodes genes related to the glycosylation process [1, 2]. Glycans, which decorate polypeptides or lipids, have essential biological functions. For example, they are involved in signal transduction, cell–cell adhesion, microbiological infections, or immune responses [3]. More and more studies describe abnormalities in glycosylation patterns as biomarkers of cancers. Osumi et al. reported that core fucosylation of E‐cadherin enhanced cell–cell adhesion of human colon carcinoma cells [4]. Another study performed by Wang et al. described a connection between abnormal UDP‐glucose dehydrogenase expression and spontaneous epithelial‐mesenchymal transition (EMT) in breast mesenchymal cell lines [5]. EMT is believed to play a crucial role in cancer progression [6, 7].

Activated forms of sugars, called nucleotide sugars, are substrates utilised in the glycosylation process. There are at least two kinds of glycosylation: one in the cytoplasm and the second in organelles, such as the Golgi apparatus (GA) or the endoplasmic reticulum. Nucleotide sugars are synthesized in the cytoplasm or nucleus [8]. In mammalian cells, two mechanisms of synthesis of nucleotide sugars exist: *de novo* and salvage. Sometimes, the same nucleotide sugar, such as GDP‐fucose, can be synthesized through both pathway types [9]. The *de novo* pathway uses GDP‐mannose as a starting point for GDP‐fucose synthesis. GDP‐D‐mannose‐4,6‐dehydratase (GMDS) and GDP‐fucose synthase (TSTA3) participate in this route. Free fucose is converted by GDP‐fucose pyrophosphorylase (FPGT) and fucokinase (FCSK) to GDP‐fucose through a salvage pathway [10]. It is estimated that about 90% of GDP‐fucose in the total pool of this nucleotide sugar in the cell is produced by the *de novo* pathway, and the rest of it is synthesized by the salvage pathway [11, 12]. Recent research by Sosicka *et al*. [13] suggested that mannose‐ and fucose‐derived GDP‐fucose co‐exist in mammalian cells in distinct pools.

Once activated, sugar has to reach the appropriate site of glycosylation: the endoplasmic reticulum (ER) or the Golgi apparatus. Nucleotide sugar transporters (NSTs) belonging to the solute carriers 35 family (SLC35) transport nucleotide sugars through membranes of the ER or the GA [14]. NSTs are type III transmembrane proteins having 6–10 transmembrane (TM) domains. All NST topologies predicted to date suggest that the C‐ and N‐termini are present on the cytosolic side, corresponding to the number of TM domains [15]. SLC35C1 (C1, GDP‐fucose transporter) protein was determined to be the leading GDP‐fucose transporter in mammalian cells [16, 17, 18]. The mutations in the gene encoding SLC35C1 protein could cause congenital disorders of glycosylation diseases, which could be moderated by fucose supplementation [19, 20, 21]. Research by our group showed that the C1 protein could discriminate between fucose‐ and mannose‐origin GDP‐fucose [22]. The SLC35C2 protein was proposed as another potential transporter of GDP‐fucose [23]. However, our group found that the simultaneous inactivation of genes encoding the SLC35C1 and SLC35C2 proteins did not influence GDP‐fucose transport across the Golgi apparatus membranes [22]. Recently, this was supported by the research of Lu *et al*. [24].

In this study, we examined the influence of inactivation of the gene encoding the SLC35C1 transporter at the level of enzymes of *de novo* and salvage biosynthesis pathways of GDP‐fucose. By double inactivation of genes encoding the SLC35C1 transporter and FCSK, GMDS, and TSTA3 proteins in various combinations, we evaluated the impact of such deficiencies on the production of nucleotide sugar and fucosylated glycan structures. We found that deficiency of the SLC35C1 protein caused a decrease/increase in FCSK, GMDS, FPGT, and TSTA3 protein levels. Importantly, we observed that the lack of C1 and some enzymes of biosynthesis pathways of GDP‐fucose stimulated incredible production of that nucleotide sugar upon fucose supplementation, and it is dependent on the deficiency of a particular enzyme constituting part of the same biosynthesis pathway. Finally, our study showed that, despite the depletion of the C1 transporter and some sources of GDP‐fucose, nucleotide sugar is delivered to the Golgi apparatus differently.

## Materials and methods

### Cell cultures and generation of knockouts

The HEK293T cell line obtained from American Type Culture Collection (ATCC, cat. num. CRL‐3216) was cultured in Dulbecco's Minimum Eagle Medium (DMEM High Glucose, Biowest, Nuaillé, France) supplemented with 10% fetal bovine serum, 100 U·mL^−1^ penicillin, and 100 μg·mL^−1^ streptomycin. Cell cultures were kept at 5% CO_2_ and a temperature of 37 °C.

Double knockouts were generated using a ready‐to‐use CRISPR/Cas9 system purchased from Santa Cruz Biotechnology (Dallas, TX, USA). To obtain cell lines deficient in SLC35C1 and fucokinase, SLC35C1 and GMDS, or SLC35C1 and TSTA3, a mixture of human FCSK double nickase plasmids (sc‐409449‐NIC), human GMDS double nickase plasmids (sc‐410794‐NIC) or human TSTA3 double nickase plasmids (sc‐408777‐NIC) was transfected into the previously described single knockout cell line C1KO HEK293T [22] according to the manufacturer's instructions. To generate a cell line lacking both fucokinase and GMDS enzymes, a mixture of human GMDS double nickase plasmids (sc‐410794‐NIC) was transfected into the previously published cell line FCSKKO HEK293T [25], following the manufacturer's instructions. For transfection, FUGENE HD transfection reagent (Promega, Madison, WI, USA) was used according to the provided protocol. After transfection, clones were isolated, and a deficiency of proteins encoded by inactivated genes was confirmed by western blotting using appropriate antibodies.

### Western blotting

Collected cells were lysed and subjected to SDS/PAGE electrophoresis on 10% polyacrylamide gels. After electrophoresis, resolved proteins were transferred onto nitrocellulose membranes (Whatman, Clifton, NJ, USA). Membranes were cut, blocked in a 5% skim milk solution in PBST buffer, and incubated with appropriate primary antibodies diluted in the SignalBoost Immunoreaction Enhancer Kit (Sigma‐Aldrich, St. Louis, MO, USA). After that, membranes were washed five times using the blocking solution and incubated with secondary antibodies diluted either in the blocking solution or the SignalBoost Immunoreaction Enhancer Kit again. Primary and secondary antibodies are listed in Table S1. Then, membranes were washed five times in the blocking solution. The chemiluminescence signal was developed using SuperSignal West Atto Ultimate Sensitivity Substrate (Thermo Fisher Scientific, Waltham, MA, USA) and visualized using the chemidoc imaging system (Bio‐Rad Laboratories, Hercules, CA, USA). Ponceau S staining or western blotting using an anti‐HSP60 antibody was performed as a loading control.

### *N*‐glycans isolation and analysis of α‐1‐6 fucosylation

Harvested cells, supplemented or not supplemented with fucose, were subjected to *N*‐glycans isolation and labelling. Briefly, the protein total pool was extracted from cell lysates and deglycosylated with *N*‐glycosidase F, then purified on graphite SPE columns, dried, and labelled with 2‐amidobenzamide (2‐AB). Then, glycan pools were digested with the mixture of neuraminidase, galactosidase, and *N*‐acetylglucosaminidase. Two remaining peaks, representing α1‐6 fucosylated *N*‐glycans and non‐fucosylated glycans, were separated on a normal phase amide column connected to the HPLC system. *N*‐glycan fucosylation was calculated by comparison of peak areas of fucosylated and non‐fucosylated truncated structures. Details of the whole procedure were described previously [22]. Purified and labelled *N*‐glycans were also subjected to MALDI‐TOF analyses as described earlier [26].

### Isolation of *O*‐glycans

Fucose‐supplemented and ‐unsupplemented cells were incubated in Dulbecco's Minimum Eagle Medium (DMEM High Glucose, Biowest) with the addition of 5% fetal bovine serum, 100 U·mL^−1^ penicillin, and 100 μg·mL^−1^ streptomycin and a peracetylated *O*‐glycan precursor (Ac_3_GalNAcBn) for 3 days. After that, a medium containing secreted *O*‐glycans was collected. Glycans were purified according to a method described by Kudelka *et al*. [27]. Briefly, *O*‐glycans were separated from the impurities, such as lipids or proteins, by membrane filtration using Amicon^®^ Ultra Centrifugal Filter, 10 kDa MWCO (Merck, Rahway, NJ, USA). Then, the filtrates containing *O*‐glycans were subjected to solid phase extraction using Supelclean™ LC‐18 SPE Tubes (Sigma‐Aldrich). Firstly, the bead was primed with acetonitrile and then washed with 0.1% trifluoroacetic acid. Next, the filtrates were applied onto columns. The column was washed with 0.1% trifluoroacetic acid. *O*‐glycans were eluted by using 50% acetonitrile with 0.1% trifluoroacetic acid and freeze‐dried under vacuum. Dried *O*‐glycans were permethylated. The excess of permethylation mixture was extracted using water and chloroform. The purified *O*‐glycan pools were subjected to MALDI‐TOF analysis.

### MALDI‐TOF analysis

Before MALDI‐TOF analysis, *N*‐glycans labelled with 2‐AB were desialylated using α2‐3,6,8,9‐neuraminidase A (New England Biolabs, Ipswich, MA, USA) and purified in the manner reported previously [26]. MALDI‐TOF experiments were performed on an Axima Performance TOF spectrometer (Shimadzu Biotech, Kyoto, Japan) equipped with a nitrogen laser (337 nm). The pulsed extraction ion source accelerated the ions to a kinetic energy of 20 keV. The data were obtained in a positive‐ion linear mode. The calibration of the linear mode analysis was done using polyethylene glycol in mass range up to 5000 Da. The accuracy of the product ion calibration is ~ 1.5 Da. The mass calibration was conducted based on the average masses. The samples were dissolved in 20% acetonitrile in water. As a matrix, 2,5‐dihydroxybenzoic acid (20 mg·mL^−1^) dissolved in 20 mm sodium acetate in 20% acetonitrile in water was applied. The sample and matrix were mixed at a 1 : 1 ratio. The resulting solution (1 mL) was spotted on a 384‐well MALDI‐TOF plate, followed by evaporation of the solvent at ambient temperature without any assistance. Each mass spectrum was accumulated from at least 200 laser shots and processed by biotech launchpad version 2.9.1 program (Shimadzu). The composition of *O*‐ and *N*‐glycans was estimated using the glycoworkbench tool (2.1) and based on the work of Kudelka *et al*. [27].

### Analysis of nucleotide sugars

Cells treated and untreated with fucose were collected, counted, and frozen. Then, cell pellets were lysed by sonication, and nucleotide sugars were isolated using the method presented previously [22].

### l‐fucose supplementation

Cells were cultured in complete media, which additionally contained a 5 mm concentration of l‐fucose for 24 h under 5% CO_2_ and a temperature of 37 °C. Conditions of fucose supplementation were adopted from our previous study [22].

### Statistical analysis

Statistical analyses were performed using graphpad prism 8 (Boston, MA, USA). One‐way or two‐way ANOVA was used to analyse data with the Tukey *post hoc* test. The percentage of fucosylation structures and concentration of GDP‐fucose, as well as relative protein levels, were analysed in three biological replicates, except the concentration of GDP‐fucose and percentage of fucosylated structures upon different fucose amounts supplementation, which were analysed in three technical replicates. Statistical parameters, including data plotted (mean ± SEM) and *P* values, are detailed in figure legends.

## Results

### Protein levels of fucose‐ and mannose‐derived GDP‐fucose biosynthesis pathway enzymes are changed in cell lines deficient in the SLC35C1 transporter

**Fig. 1:**
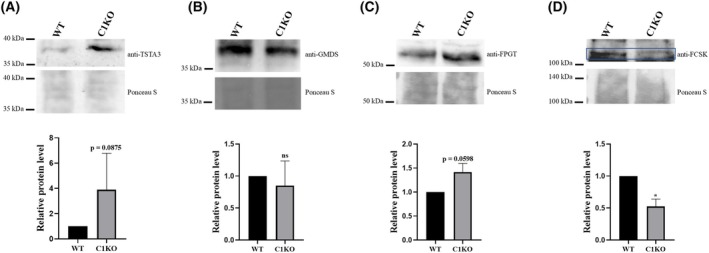
Estimation of TSTA3, GMDS, FPGT, and FCSK protein levels in HEK293T C1KO cell line. C1KO cell line was subjected to western blotting of (A) TSTA3, (B) GMDS, (C) FPGT, and (D) FCSK and compared with the wild‐type cell line. Ponceau S staining was used as a loading control for all western blotting experiments. Data are presented as mean ± SEM. Each sample was run in three biological replicates (*n* = 3). ns, not statistically significant; *, *P* < 0.05 as determined using one‐way ANOVA with the Tukey *post hoc* test. *P* values trending to be statistically significant are also shown. The blue frame indicates the signal from FCSK protein.

Previously, our group found that the GDP‐fucose transporter SLC35C1 might preferentially transport GDP‐fucose depending on its origin. SLC35C1 may be responsible for delivering GDP‐fucose produced from GDP‐mannose to a greater extent and less from the salvaged one [19]. Therefore, we decided to investigate whether the absence of the C1 transporter would influence the protein level of particular enzymes of GDP‐fucose biosynthesis pathways. We used the HEK293T cell line deficient in SLC35C1 for that study. We observed slightly elevated TSTA3 and FPGT protein levels in C1KO cells (Fig. 1A,C) compared to wild‐type cells. The protein level of GMDS was not changed in C1KO cells compared to wild‐type cells (Fig. 1B). Compared to wild‐type cells, the level of FCSK enzyme was reduced (Fig. 1D). Those results showed that the absence of the C1 transporter raised the levels of enzymes that are responsible for creating the end product, GDP‐fucose.

### Generation of double knockouts in the HEK293T cell lines

Having found that a relationship between the GDP‐fucose transporter and enzymes of the *de novo* and salvage biosynthesis pathways may exist, we generated cell lines deficient in the SLC35C1 protein and selected enzymes of GDP‐fucose biosynthesis pathways in the HEK293T cell line. Applying the CRISPR/Cas9 method, we generated a cell line deficient in the SLC35C1 and TSTA3 proteins simultaneously (C1TSTA3KO), a cell line deficient in the SLC35C1 and GMDS proteins simultaneously (C1GMDSKO) and a cell line deficient in the SLC35C1 and FCSK proteins simultaneously (C1FCSKKO). To do this, we used the previously described cell line lacking SLC35C1 proteins, HEK293T C1KO [22]. The absence of proteins was confirmed by western blotting (Fig. S1A–C). Additionally, as a control, we generated a cell line deprived of all sources of GDP‐fucose, deficient in FCSK and GMDS (FCSKGMDSKO), using the previously described FCSKKO HEK293T cell line [25]. Western blotting was done to verify the absence of FCSK protein (Fig. S1D).

### Analysis of the intracellular concentration of GDP‐fucose and the amount of α‐1‐6 fucosylated structures revealed unequal delivery of nucleotide sugar to the Golgi apparatus

**Fig. 2:**
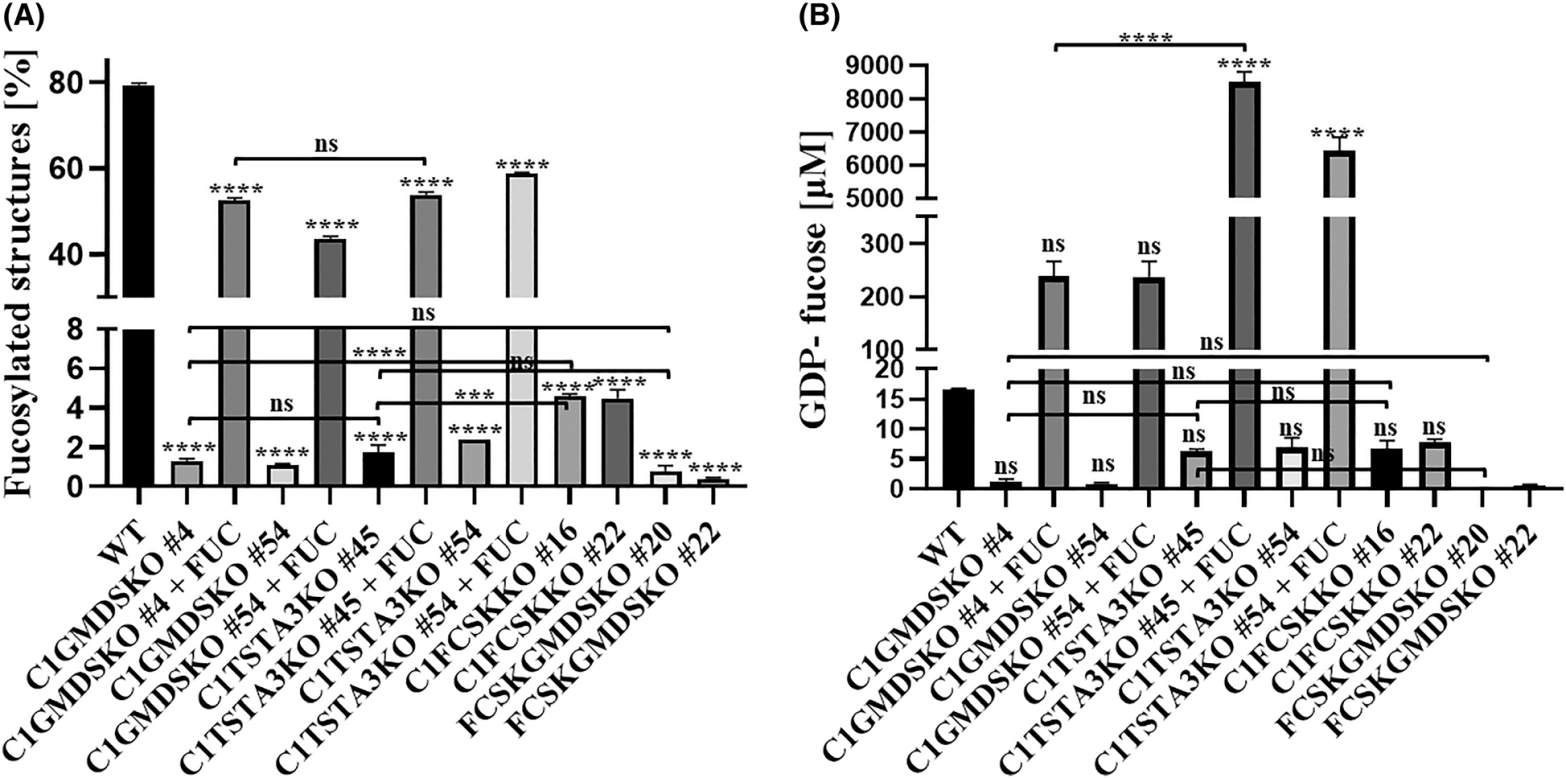
Analysis of the amount of fucosylated structures and GDP‐fucose concentration in double knockout and wild‐type cell lines. (A) *N*‐glycans from fucose‐treated and untreated cells were isolated, fluorescently labelled and treated with the mixture of specific exoglycosidases. Then, two peaks representing alpha 1–6 fucosylated and not‐fucosylated structures were separated on an amide column, connected to HPLC system [19]. (B) Intracellular concentration of GDP‐fucose extracted from double knockouts and wild‐type cell lines supplemented and unsupplemented with fucose. Concentration of GDP‐fucose was calculated after purification of cytosolic nucleotide sugars on SPE porous graphite columns followed by HPLC ion‐pairing chromatography. A typical chromatograms of ion‐pairing chromatography separations of nucleotides extracted from WT and selected double mutants supplemented with external fucose and not treated are present in Fig. S2. Data are presented as mean ± SEM. Each sample was run in three biological replicates (*n* = 3), ns, not statistically significant, ***, *P* < 0.001 and ****, *P* < 0.0001, as determined using one‐way ANOVA with the Tukey *post hoc* test.

To study the GDP‐fucose transporter's role in delivering GDP‐fucose originating from various sources of this nucleotide sugar, we applied generated double knockouts to examine the amount of α‐1‐6 fucosylated structures by applying glycosidase digestion of *N*‐glycans isolated from whole cells coupled with HPLC separation [22]. Deficiency of the SLC35C1 transporter and GMDS enzyme significantly decreased the level of α‐1‐6 fucosylated structures from ~ 80% in wild‐type cells (Fig. 2A) to ~ 1.3% (Fig. 2A) in C1GMDSKO cells. This level increased to ~ 40–50% upon fucose supplementation, but it was still not equal to the amount obtained in the wild‐type cell line. Almost the same result was gained from cells lacking the SLC35C1 protein and TSTA3 enzyme (Fig. 2A). The amount of α‐1‐6 fucosylated structures in non‐treated cells was ~ 2% and, upon supplementation, increased to ~ 53–58%. No significant difference in the level of α‐1‐6 fucosylated structures was found between the C1GMDSKO and C1TSTA3KO cell lines, either untreated or treated with fucose. Interestingly, the level of α‐1‐6 fucosylated structures in the C1FCSKKO cell line was higher (~ 4%, Fig. 2A) than in the C1GMDSKO and C1TSTA3KO cell lines. Previously, we reported on potential routes of delivery of GDP‐fucose to the GA, where the SLC35C1 transporter transported GDP‐fucose originating mainly from the *de novo* biosynthesis pathway [22]. Here, it was observed that more nucleotide sugar passed through to the GA in the absence of the SLC35C1 protein and FCSK enzyme when only GDP‐fucose produced by the *de novo* biosynthesis pathway was available. However, the difference was relatively small, only ~ 2‐fold, between the C1FCSKKO cell line and the C1TSTA3/GMDSKO cell lines.

Before the nucleotide sugar is incorporated into the oligosaccharide structure, it must be produced on the cytosolic side of the GA/ER membrane. Therefore, we examined the concentration of GDP‐fucose in generated double knockouts, using ion‐pairing chromatography (Fig. S2). The concentration of nucleotide sugar isolated from the C1GMDSKO cells dropped from ~ 16.5 μm in the wild‐type cells to ~ 1 μm (Fig. 2B). Upon fucose supplementation, the concentration of GDP‐fucose increased to ~ 240 μm and was comparable to the concentration obtained in the wild‐type cells fed with fucose [22]. A smaller decrease was observed in the C1TSTA3KO cell line. In cells not treated with fucose, the concentration of GDP‐fucose was around 6–7 μm (Fig. 2B) and unexpectedly increased to ~ 6000–8000 μm (~ 1000‐fold) upon external fucose supplementation. Previously, we reported abnormal production of GDP‐fucose in cells lacking only the TSTA3 protein upon fucose supplementation. The concentration of GDP‐fucose in those cells was around 2.0 mm [25]. Herein, that effect seemed even more promoted in the absence of the SLC35C1 protein because the fold difference between TSTA3KO cells and C1TSTA3KO cells was increased about 3–4 times. Interestingly, the concentration of GDP‐fucose in C1FCSKKO cells dropped from ~ 16.5 μm in wild‐type cells to ~ 6 μm (Fig. 2B). It is an extraordinary finding, as it is claimed that about 90% of the total GDP‐fucose pool in mammalian cells is produced via the *de novo* biosynthesis pathway [11, 12]. The production of nucleotide sugar would probably be associated with the availability of its transporting route. On the other hand, we found that in the absence of only the SLC35C1 protein, there were no disturbances in GDP‐fucose production under normal conditions [22]. In the control cell line FCSKGMDSKO, where both GDP‐fucose synthesis pathways are blocked, the concentration of GDP‐fucose was not detectable (the detection level of the UV‐diode array was around 0.03–0.5 μm, Fig. 2B).

### Differential effect of various fucose concentrations on GDP‐fucose synthesis in C1GMDSKO and C1TSTA3KO cell lines

Previously, we observed various responses, in terms of GDP‐fucose production, of cell lines deficient in TSTA3 and GMDS enzymes to different fucose concentrations [25]. Herein, having found considerable differences in the production of GDP‐fucose by C1GMDSKO and C1TSTA3KO double KO cells, even more prominent than in the case of single inactivation of genes encoding enzymes of the *de novo* GDP‐fucose biosynthesis pathway, we wondered whether they would stay constant through applying different fucose concentrations in culture media.

**Fig. 3:**
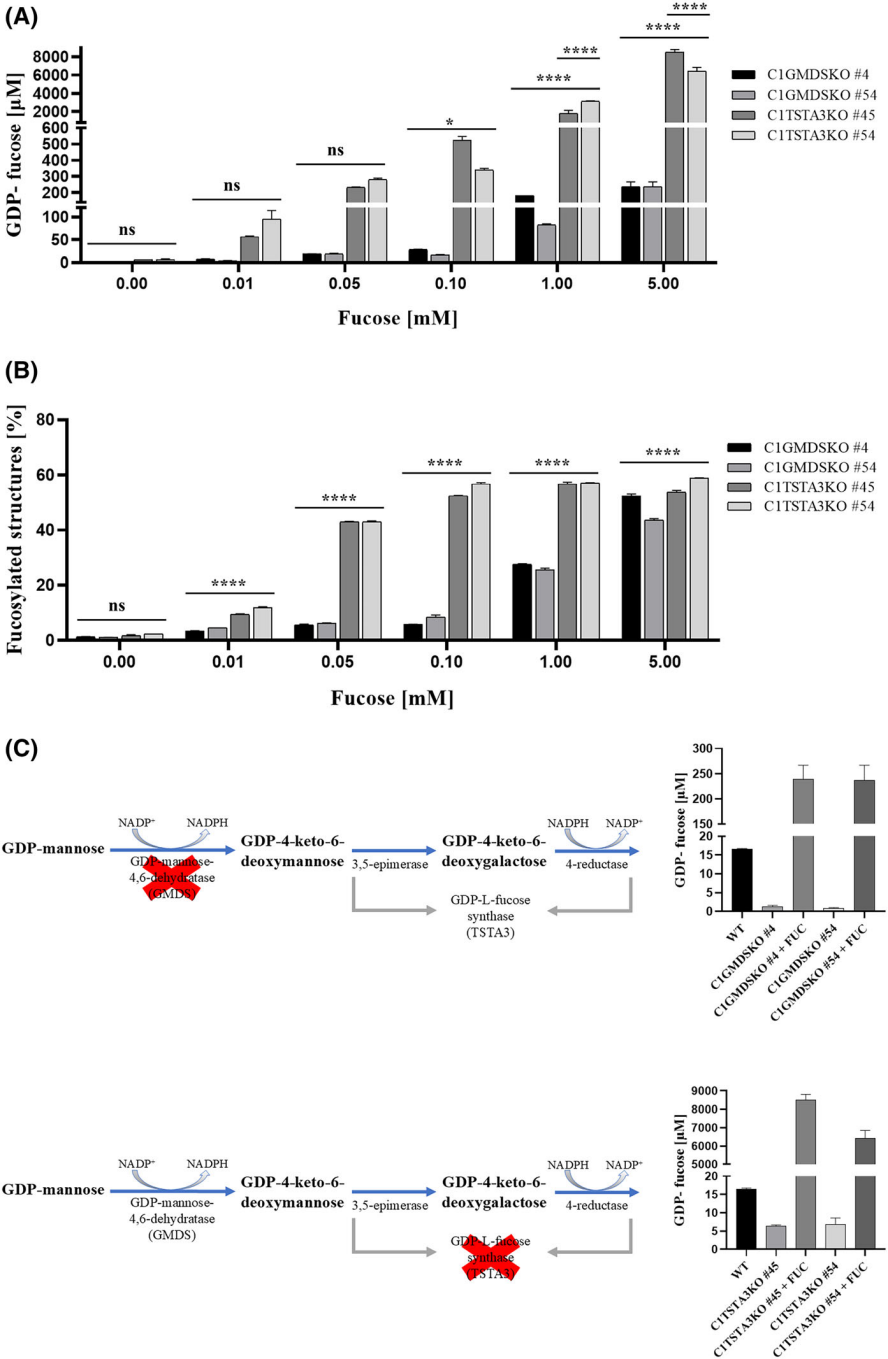
Analysis of percentage of fucosylated structures and intracellular concentration of GDP‐fucose in C1GMDSKO and C1TSTA3KO cell lines. (A) From cells supplemented with different fucose concentrations. Nucleotide sugars were extracted and separated in HPLC. GDP‐fucose concentration was calculated based on the method described previously [19]. (B) Cells were treated with different fucose concentrations, and then *N*‐glycans were isolated and subjected to HPLC. The percentage of fucosylated structures was calculated based on the method described previously [23]. Each experiment was performed once (*n* = 1), but aliquots taken from partially purified samples were examined in chromatography, in three independent, technical replicates, ns, not statistically significant, *, *P* < 0.05 and ****, *P* < 0.0001, as determined using one‐way ANOVA with the Tukey *post hoc* test. (C) Presentation of main changes in GDP‐fucose production upon inactivation of specific *de novo* enzyme. Data are presented as mean ± SEM.

Analysis of the concentration of nucleotide sugar revealed considerable differences in the production of GDP‐fucose between C1TSTA3KO and C1GMDSKO cell lines. Supplementation with a 10 μm concentration of fucose in media already increased the concentration of nucleotide sugar in C1TSTA3KO cells from ~ 6 to 7 μm, at 0 concentration of fucose in cell culture media, to ~ 50–90 μm (Fig. 3A). Meanwhile, the change in C1GMDSKO was barely visible, from ~ 1 μm at 0 concentration of fucose in cell culture media to ~ 4–7 μm (Fig. 3A). Those differences remained consistent throughout all applied fucose concentrations. At least a 10‐times difference was maintained between GDP‐fucose concentration in C1TSTA3KO cells and C1GMDSKO cells. The main inconsistency was observed for the 5 mm concentration of applied fucose, where for C1GMDSKO the GDP‐fucose concentration rose to ~ 230 μm, while for C1TSTA3KO cells that concentration was ~ 6–8 mm (Fig. 3A).

In the case of incorporation of the produced nucleotide sugar, the amount of α‐1‐6 fucosylated structures was also examined. The level of fucosylated structures in C1GMDSKO cells was similar to that in wild‐type cells only upon 5 mm fucose supplementation (Fig. 3B). On the other hand, for C1TSTA3KO cells, that level was noted for 0.1 mm fucose concentration (Fig. 3B). For C1GMDSKO cells, only after application of 1 mm fucose concentration in cell culture media did the amount of α‐1‐6 fucosylated structures start to increase, in contrast to the C1TSTA3KO cell line, where application of 10 μm fucose caused a higher level of fucosylated oligosaccharides. The main effect in GDP‐fucose production upon inactivation of specific *de novo* enzyme was presented in Fig. 3C.

### Structural analysis of *N*‐glycans

**Fig. 4:**
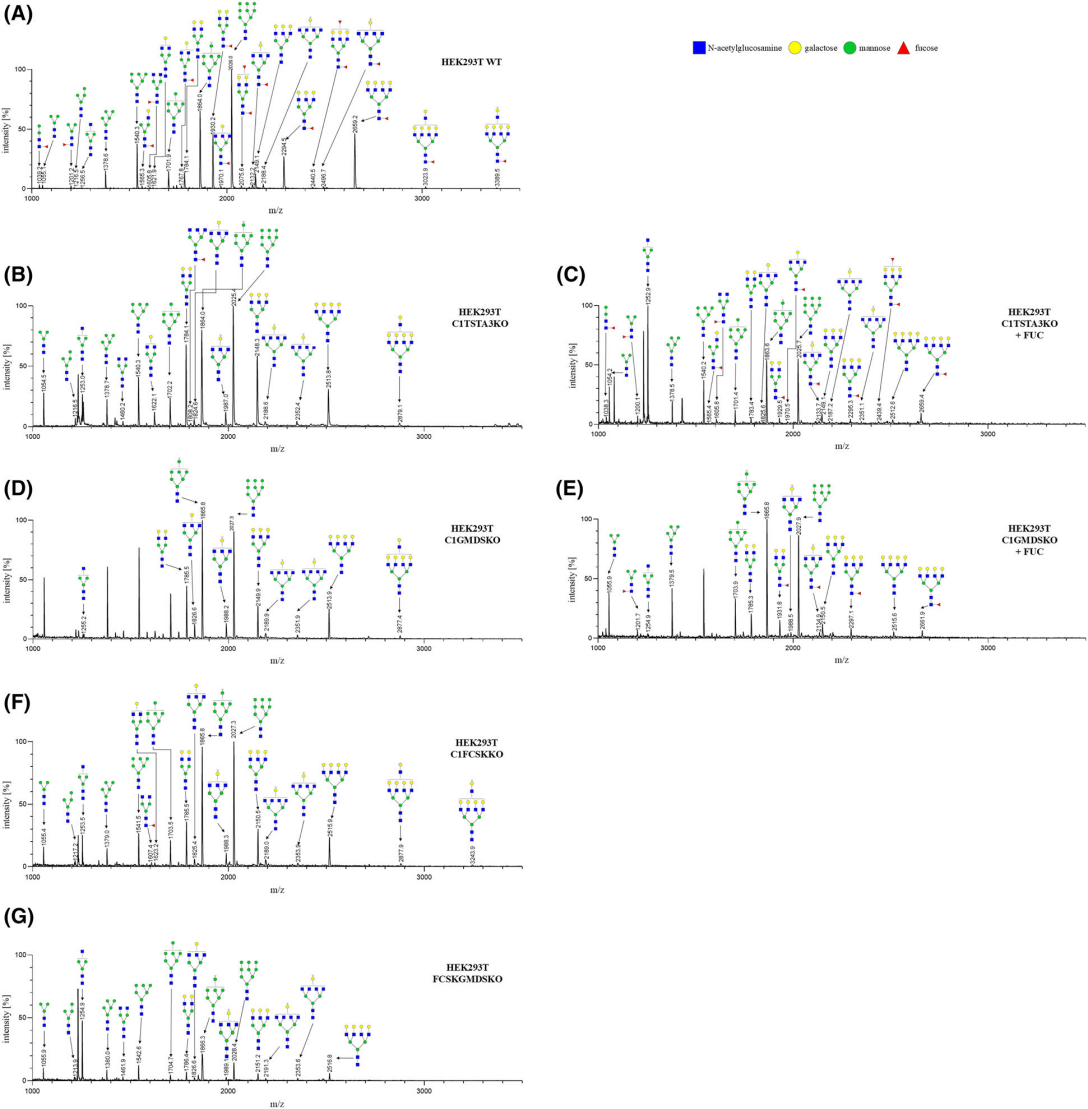
Presentation of *N*‐glycan structures isolated from wild‐type and double knockout cell lines. Extracted *N*‐glycans from (A) wild‐type cell line; (B) and (C) C1GMDSKO cell line, treated and not treated with fucose; (D) and (E) C1TSTA3KO cell line, supplemented with fucose and not; (F) C1FCSKKO cell line; and (G) FCSKGMDSKO cell line were subjected to MALDI‐TOF analysis in positive‐ion mode and detected as [M + Na]^+^. Oligosaccharide structures were revealed using the glycoworkbench tool (2.1). Blue squares, GlcNAc; green circles, mannose; yellow circles, galactose; red triangles, fucose. All MALDI‐TOF data (A–G) were recorded once (*n* = 1).

For a more in‐depth study of the impact of double knockouts on glycosylation, we carried out a structural analysis of *N*‐glycans. Depletion of TSTA3 and the SLC35C1 proteins simultaneously did not disturb the synthesis of non‐fucosylated immature *N*‐glycans or complex ones (Fig. 4B) compared to *N*‐glycans isolated from the wild‐type cells (Fig. 4A). However, it strongly influenced fucosylation of *N*‐glycans, because only one fucosylated species appeared, non‐galactosylated tri‐antennary, ~ m/z 1808.2. This observation could show that not only the fucosylation pattern has changed but also the galactosylation pattern. Upon fucose supplementation, fucosylated species were almost completely restored (Fig. 4C), and the glycosylation pattern was highly similar to that of wild‐type cells. The opposite was found for *N*‐glycans isolated from C1GMDSKO cells (Fig. 4D). No fucosylated structures were observed. Moreover, double inactivation of genes encoding the SLC35C1 and GMDS proteins caused disturbances in the synthesis of immature *N*‐glycans compared to *N*‐glycans isolated from the wild‐type cells. The synthesis of tri‐ and tetra‐antennary *N*‐glycans was more promoted. Even after supplementation of C1GMDSKO cells with fucose (Fig. 4E), not all fucosylated species appeared compared to the glycosylation pattern in the wild‐type cells. The differences in detected fucosylated structures between fucose‐unsupplemented C1GMDSKO and C1TSTA3KO cells may result from the lower level of α‐1‐6 fucosylated structures examined earlier. The diversity of *N*‐glycans extracted from C1FCSKKO cells highly resembled *N*‐glycan species isolated from C1TSTA3KO cells (Fig. 4F). Compared to the fucosylation pattern of glycans of wild‐type cells, no fucosylated species were detected in C1FCSKKO cells, except for one, non‐galactosylated bi‐antennary, which was not present in wild‐type cells, ~ m/z 1607.4. There is another reason to make assumptions that not only the fucosylation pattern has changed but also the galactosylation pattern. No fucosylated species were noted in FCSKGMDSKO cells, which constituted a control (Fig. 4G) compared to wild‐type cells. Also, no impact on synthesizing other kinds of *N*‐glycans was noted. Intriguingly, the lack of GMDS protein in a companion to the deficiency of the SLC35C1 protein has influenced the synthesis of immature bi‐antennary *N*‐glycans. Meanwhile, the complete absence of GDP‐fucose in cells did not cause similar effects.

### Structural analysis of *O*‐glycans

**Fig. 5:**
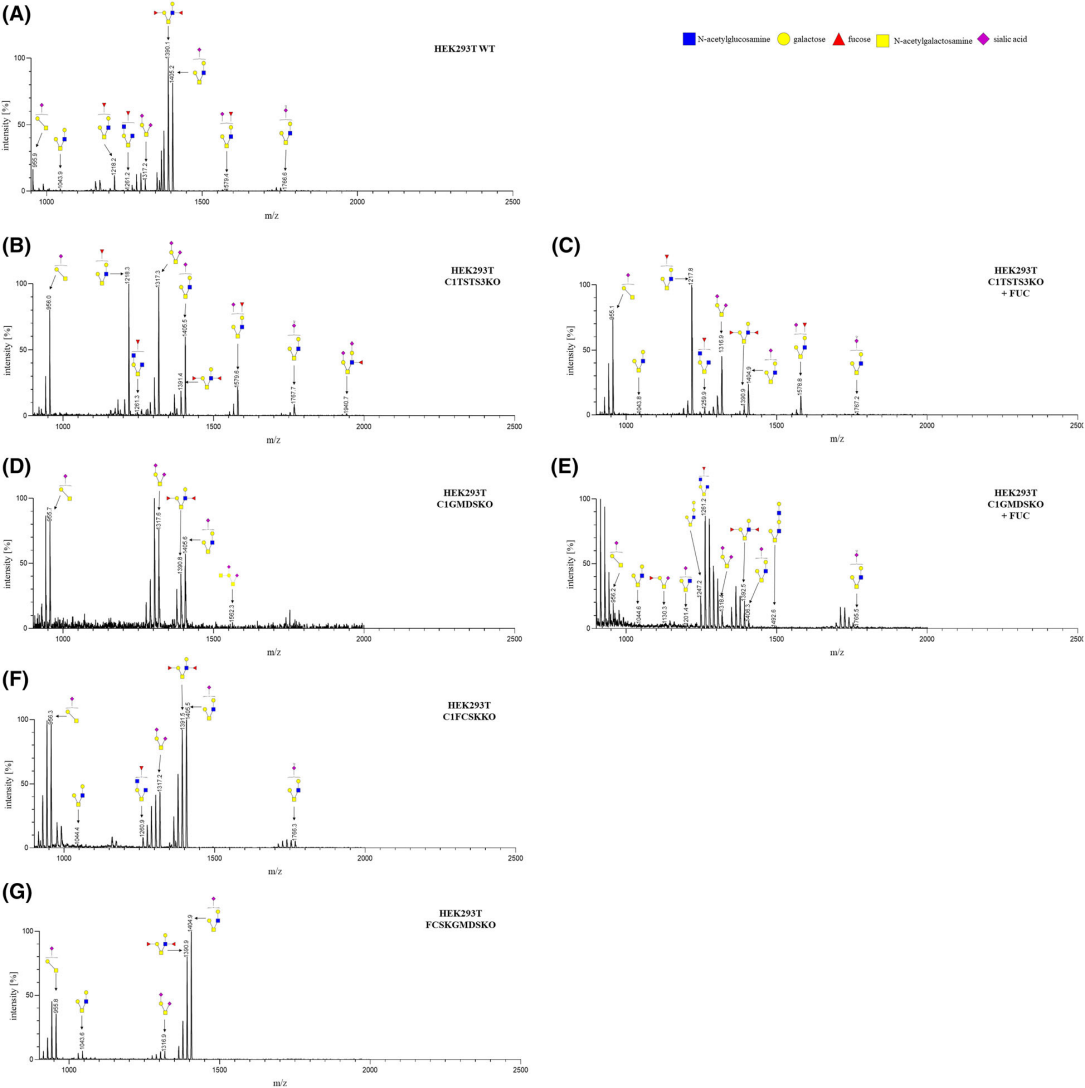
Presentation of *O*‐glycan structures isolated from wild‐type and double knockout cell lines. Extracted *O*‐glycans from (A) wild‐type cell line; (B) and (C) C1GMDSKO cell line, treated and not treated with fucose; (D) and (E) C1TSTA3KO cell line, supplemented with fucose and not; (F) C1FCSKKO cell line; and (G) FCSKGMDSKO cell line were subjected to MALDI‐TOF analysis in positive‐ion mode and detected as [M + Na]^+^. O‐glycan composition was estimated using the glycoworkbench tool (2.1) and the published work of Kudelka *et al*. [27]. Blue squares, GlcNAc; yellow squares, GalNAc; yellow circles, galactose; red triangles, fucose; pink diamonds, sialic acid. All MALDI‐TOF data (A–G) were recorded once (*n* = 1).

Structural analysis of *O*‐glycans isolated from C1TSTA3KO cells revealed a smaller impact of genetic manipulation than in the case of *N*‐glycans. Compared to the *O*‐glycans extracted from wild‐type cells (Fig. 5A), all fucosylated species were detected among *O*‐glycans isolated from TSTA3C1KO cells (Fig. 5B). Even new species appeared (~ m/z 1940.7). There is almost no difference between glycans identified in unsupplemented C1TSTA3KO cells and those supplemented with fucose (Fig. 5C). On the other hand, *O*‐glycans obtained from C1GMDSKO cells were significantly affected (Fig. 5D). There were fewer fucosylated species compared to O‐glycans found in wild‐type cells. However, depletion of the SLC35C1 transporter and GMDS protein simultaneously had a milder effect on *O*‐glycans than on *N*‐glycans. Fucose supplementation enormously improved the diversity of *O*‐glycans' structures in C1GMDSKO cells. More importantly, new fucosylated species appeared, even ones that were not detected among isolated structures from wild‐type cells (Fig. 5E). Comparing the fucose supplementation effect on *O*‐glycan structures and species isolated from cells not supplemented with fucose, it is evident that the absence of GMDS coupled with deficiency of the SLC35C1 protein had a more significant negative impact on fucosylation and overall glycosylation than the double inactivation of genes encoding TSTA3 and the SLC35C1 transporter. The simultaneous absence of FCSK and the SLC35C1 transporter also decreased the number of fucosylated *O*‐glycans (Fig. 5F), especially α‐1‐2 fucosylated ones, compared to *O*‐glycans isolated from wild‐type cells or C1TSTA3KO cells. Still, that change was less severe than in C1GMDSKO cells. Interestingly, in a control cell line, the FCSKGMDSKO cell line, some residual fucosylated species were detected (Fig. 5G), which were not observed in the case of the structural analysis of *N*‐glycans. This cannot be simply explained by contamination of glycans from cell culture media because of the unique procedure of biosynthesis of *O*‐glycans secreted to the media and purified according to the CORA technique. Probably, there is some residual, very low (nanomolar) level of GDP‐fucose still present, even in cells with no functional pathways of GDP‐fucose biosynthesis. However, it was not proved in this report; the detection level of intracellular concentration of the metabolite after chromatographic separation of the nucleotide pool was approximately in the range of 0.2–0.5 μm.

Previously, we proposed three routes of delivery of GDP‐fucose produced from distinct sources to the Golgi apparatus [22]: first, SLC35C1‐dependent, which mainly utilizes mannose‐derived GDP‐fucose; second, C1‐independent, which transports primarily the nucleotide sugar synthesized from fucose and works under very low (nanomolar) concentrations of exogenous fucose; and third, also C1‐independent, which utilizes fucose‐derived GDP‐fucose, although this route requires much higher concentrations of this nucleotide sugar that can only be obtained by feeding the cells with sub‐millimolar and millimolar concentrations of fucose. Results obtained from the structural analysis of glycans provide evidence for this hypothesis. Moreover, they may show that particular routes probably deliver GDP‐fucose to individual stacks of the Golgi apparatus because different fucosyl bonds are not equally affected by different combinations of inactivated genes encoding the C1 transporter and enzymes of GDP‐fucose biosynthesis pathways, due to the hypothetical existence of various fucosyltransferases in different Golgi apparatus stacks [28, 29].

## Discussion

In this study, we examined the influence of the lack of the SLC35C1 protein on the production of GDP‐fucose, coupled with the absence of particular sources of GDP‐fucose. Moreover, we assessed the incorporation of fucose into oligosaccharides and discovered a differential impact on the production of fucosylated *O*‐ or *N*‐glycans, even if simultaneous inactivation of genes encoding the SLC35C1 protein and enzymes of the biosynthesis pathway of GDP‐fucose belonged to the same biosynthesis pathway.

The glycosylation process mainly occurs in the Golgi apparatus, and the synthesis of nucleotide sugars (NS), substrates in this process, takes place in the cytoplasm or nucleus [8, 30]. Therefore, some transporting mechanisms should exist for efficient delivery of nucleotide sugars, at least in the membranes of GA and/or ER. The SLC35C1 was described as a GDP‐fucose transporter [15, 16, 17]. However, it seems that GDP‐fucose may be translocated to GA in three different ways: (a) dependent on the SLC35C1 transporter; (b) C1‐independent, which works at a very low, nanomolar concentration of fucose feeding; and (c) also C1‐independent, requiring a much higher, millimolar or submolar concentration of supplemented fucose [22]. To date, there are only some reports about the positive correlation between high SLC35C1 mRNA levels and the mRNA levels of enzymes of the *de novo* GDP‐fucose biosynthesis pathways [31, 32]. When studying the effect of the deficiency of the SLC35C1 transporter on protein levels of FCSK, TSTA3, FPGT, and GMDS enzymes, we obtained a differential effect. Compared to the levels of TSTA3 and FPGT proteins detected in wild‐type cells, the deficiency of the SLC35C1 protein resulted in a slight elevation of their levels (Fig. 1A,C). Unexpectedly, the absence of the C1 protein caused a slight reduction in the level of the FCSK enzyme (Fig. 1D). It was reported that the FCSK enzyme could be inhibited by GDP‐fucose [33]. Therefore, elevated levels of two enzymes responsible for the last step of GDP‐fucose biosynthesis could influence the protein level of FCSK. However, it was also found that GMDS could be inhibited by GDP‐fucose [34]. In the case of GMDS, its level did not change in C1KO cells compared to wild‐type cells (Fig. 1B).

We studied the influence of the simultaneous deficiency of the SLC35C1 transporter and the chosen enzymes of GDP‐fucose biosynthesis pathways on GDP‐fucose synthesis and its incorporation into oligosaccharides by the residual pathway of GDP‐fucose synthesis. To do that, we applied the CRISPR/Cas9 approach (Fig. S1A–D). Additionally, the double inactivation of GMDS and FCSK in the HEK293T cell line was performed as a control, in which no nucleotide sugar synthesis would be obtained (Fig. S1D).

In the double knockouts of the SLC35C1 and TSTA3 or GMDS enzymes, in normal conditions, a significant reduction in the level of α‐1‐6 fucosylated structures was observed (Fig. 2A), as well as a decrease in GDP‐fucose concentration (Fig. 2B), compared to the values obtained for wild‐type cells. Interestingly, upon fucose supplementation, a vast induction of GDP‐fucose synthesis was observed in C1TSTA3KO cells, with the GDP‐fucose concentration rising to about 6–8 mm (Fig. 2B). Meanwhile, that effect was not observed in C1GMDSKO cells (Fig. 2B). It was found that 60 μm of GDP‐fucose almost wholly inhibited the action of FCSK prepared from pig kidney [33]. That effect is not observed in C1GMDSKO or C1TSTA3KO cells. However, those differences in the production of GDP‐fucose, when the concentration of supplemented fucose was 5 mm, did not cause a similar effect on the incorporation of fucose into oligosaccharides (Fig. 2A). To the best of our knowledge, similar experiments have not been conducted before. Previously, we suggested mutual regulation of the action of the *de novo* and the salvage pathways of GDP‐fucose production [25]. In that study, we observed similar enhanced production of this nucleotide sugar. Conflicting information came from experimental studies performed on cell lines lacking NSTs. In native conditions, knockout of the SLC35A1 (CMP‐sialic acid transporter, A1) protein caused an increase in CMP‐sialic acid, whereas, with a deficiency of the SLC35C1 transporter, such accumulation did not occur [22, 35]. However, the supplementation of cells lacking the SLC35C1 protein with fucose increased the concentration of GDP‐fucose only when cells were supplemented with fucose having a concentration of at least 0.1 mm [22]. Also, supplementation of cells deficient in the SLC35A1 transporter with 10 mm *N*‐acetylneuraminic acid caused an increase in CMP‐sialic acid in cells. Herein, the effect of accumulation/induction seems to have been enhanced by the absence of a transporter, similarly to the study on cells lacking the A1 transporter. Possibly, it is the effect of the accumulation of NS, because it is not fully used for fucosylated glycan synthesis. Significantly, the amount of fucosylated structures in cells with double knockouts did not reach the same value as in wild‐type cells (Fig. 2A). However, it is worth noting that massive production of NS is observed in the case of double inactivation of genes encoding the SLC35C1 transporter and TSTA3 enzyme, but not in case of deficiency of the SLC35C1 transporter and GMDS. Possibly, this effect is caused by overlapping of two or more factors: lack of an efficient transporting system, impaired regulation of the action of the salvage pathway by the *de novo* pathway, which we postulated earlier, and lack of feedback inhibition of GDP‐fucose synthesis by some fucosylated proteins – as yet undiscovered regulatory factors – which are not fully fucosylated due to insufficient fucosylation efficiency. Surprisingly, the simultaneous deficiency of the SLC35C1 transporter and FCSK enzyme had less impact on the incorporation of GDP‐fucose into oligosaccharides, compared to C1TSTA3/GMDSKO cell lines (Fig. 2A). However, the concentration of GDP‐fucose in C1FCSKKO cells is highly similar to that in the C1TSTA3KO cell line (Fig. 4B). It was found that GDP‐fucose originating from mannose or glucose is a little more readily incorporated into the chitobiose core of *N*‐glycans than GDP‐fucose originating from salvaged fucose [13]. Therefore, those differences between the C1TSTA3/GMDSKO cell lines and the C1FCSKKO cell line could arise from the preferential usage of GDP‐fucose by fucosyltransferase 8 (FUT8).

The differential production of GDP‐fucose between the C1GMDSKO and C1TSTA3KO cell lines was constant even if various fucose concentrations were applied (Fig. 3A). In the case of both cell lines, GDP‐fucose synthesis was enhanced in all used fucose concentrations (Fig. 3A). For C1GMDSKO cells, the change in the production of nucleotide sugar was slight until the fucose concentration of 1 mm was used. However, the same amount of fucose used in C1TSTA3KO cells caused the increase in GDP‐fucose to 2–3 mm. That result is quite similar to those obtained for the single inactivation of genes encoding TSTA3 or GMDS enzymes [25]. However, the increase in GDP‐fucose synthesis in cells with a single inactivation of genes encoding the *de novo* pathway enzymes was smaller than in cells with a double inactivation of genes encoding the *de novo* pathway enzymes and the SLC35C1 transporter. Therefore, it raises the question of the reason for such a phenomenon. One possibility is the lack of an efficient transporting system for GDP‐fucose into the GA and the accumulation of the substrate. It seems plausible because, comparing the effect of not very enhanced GDP‐fucose production in the GMDSKO cell line and the obtained percentage of fucosylated structures, it is apparent that in the presence of the SLC35C1 transporter, the amount of fucosylated *N*‐glycans increased to almost the same level as that observed in wild‐type cells. On the other hand, after reaching the maximal value of fucosylated structures, the production of GDP‐fucose would be stopped, and the concentration of nucleotide sugar should be the same in single knockouts and double knockouts. In the case of the C1TSTA3KO and the TSTA3KO cell line, the situation was different. Upon 5 mm fucose supplementation, the GDP‐fucose concentration was 2–3 and 6–8 mm for the TSTA3KO and the C1TSTA3KO cell lines, respectively. Previously, we hypothesized about interrupting interactions between enzymes of GDP‐fucose biosynthesis pathways. Such interruption could cause abnormalities in nucleotide sugar synthesis because of the lack of physical regulation of the working of enzymes. A similar effect was previously obtained for localization studies of the yeast Gal7p protein, which is the homologue of human galactose‐1‐phosphate uridylyltransferase (GALT) in *Saccharomyces cerevisiae* [36].

Structural analysis of *N*‐ and *O*‐glycans seems to confirm previous considerations. Looking at structures of *N*‐glycans isolated from the C1TSTA3KO cells, a considerable reduction was observed in the number of fucosylated species (Fig. 4B) compared to fucosylated *N*‐glycans isolated from the wild‐type cells (Fig. 4A). Interestingly, upon fucose supplementation, not all fucosylated glycans were restored (Fig. 4C). Moreover, the antennae fucosyl bonds were observed to be more promoted in C1TSTA3KO cells when examining the *N*‐ and *O*‐glycan structures isolated from those cells (Figs 4C and 5B,C). This observation is in contrast to the hypothesis of unequal distribution of GDP‐fucose originating from different sources [13]. In that study, the authors claimed that externally added fucose is used chiefly to synthesize α‐1‐6 fucosylated glycans. Additionally, the double inactivation of genes encoding the SLC35C1 protein and GMDS enzymes significantly influenced the synthesis of immature *N*‐glycans. Despite a significant decrease in fucosylated oligosaccharides, *O*‐glycans were less disturbed than *N*‐glycans (Figs 4D and 5D). Even upon fucose supplementation, the fucosylated species, either *N*‐ or *O*‐glycans, were reduced compared to those isolated from the wild‐type cells (Figs 4E and 5E), and in comparison to oligosaccharides extracted from the C1TSTA3KO cell line (Figs 4D and 5D). In theory, fucosyltransferases (FUTs) are located in different stacks of the Golgi apparatus [28, 29]. Apparently, in the absence of mannose‐derived GDP‐fucose, the rest of this nucleotide sugar originating from the salvage biosynthesis pathway seems to be translocated near the α‐1‐2 and α‐1‐3/4 fucosyltransferases rather than close to α‐1‐6 FUT8, looking at the differential changes in fucosylation pattern of *O*‐ and *N*‐glycans in generated double knockouts. However, that effect is unequal among the C1TSTA3KO and the C1GMDSKO cell lines. The absence of GMDS or TSTA3 proteins could probably change the localization of FPGT and FCSK enzymes, resulting in an unequally distributed, locally higher concentration of produced GDP‐fucose. Furthermore, the outcome of detecting fucosylated species in the C1FCSKKO cells (Figs 4F and 5F) seems to confirm separate routes of delivering GDP‐fucose to the GA. It is a consequence of the higher amount of α‐1‐6 fucosylated structures compared to the C1GMDS/TSTA3KO cell lines, wherein the concentration of GDP‐fucose in the C1TSTA3KO and C1FCSKKO cells was almost the same (Fig. 2A,B).

Overall, the double inactivation of genes encoding the C1 protein and enzymes of *de novo* and salvage pathways impacted GDP‐fucose synthesis and then its incorporation into glycans' structures. More interestingly, that effect was unequal. The deficiency of the C1 and TSTA3 led to massive production of GDP‐fucose. Meanwhile, the deficiency of the C1 and GMDS did not. It is worth noting that GMDS and TSTA3 proteins belong to the same GDP‐fucose biosynthesis pathway, *de novo*, and are completely blocked in both double KO cells. It could mean that the C1 protein plays a regulatory role in GDP‐fucose production and has a direct or indirect influence on the alternative salvage pathway of GDP‐fucose biosynthesis. Our study also showed that the deficiency of the SLC35C1 protein could influence the protein level of GDP‐fucose synthesis enzymes, especially those responsible for the final steps of biosynthesis pathways, FPGT and TSTA3 enzymes.

## Conflict of interest

The authors declare no conflict of interest.

## Peer review

The peer review history for this article is available at https://www.webofscience.com/api/gateway/wos/peer‐review/10.1002/2211‐5463.70057.

## Author contributions

ES: conceptualization, methodology, formal analysis, validation, investigation, writing – original draft, visualization, funding acquisition. MO: conceptualization, methodology, investigation, writing – review & editing, funding acquisition.

## Supporting information


**Fig. S1.** Confirmation of gene inactivation in double knockout generated in HEK293T cell line.
**Fig. S2.** An example of the separations of partially purified nucleotide sugar polls, extracted from selected cell lines.
**Table S1.** List of antibodies used in western blotting analysis.

## Data Availability

The data that supports the findings of this study are available in the Supporting Information of this article. The MS data has been deposited in GlycoPost under ID GPST000574. The chromatography data are deposited in Dryad (DOI: 10.5061/dryad.95x69p8w5).
