# Supplementary material for: GDP‐fucose transporter SLC35C1: a potential regulatory role in cytosolic GDP‐fucose and fucosylated glycan synthesis
Source: FEBS Open Bio. 2025 May 27;15(8):1336–49. doi: 10.1002/2211-5463.70057 (PMC12319708; doi:10.1002/2211-5463.70057)
Supplement: Supplementary file 1 — Fig. S1. Confirmation of gene inactivation in double knockout generated in HEK293T cell line. Fig. S2. An example of the separations of partially purified nucleotide sugar polls, extracted from selected cell lines. Table S1. List of antibodies used in western blotting analysis. [file FEB4-15-1336-s001.pdf]

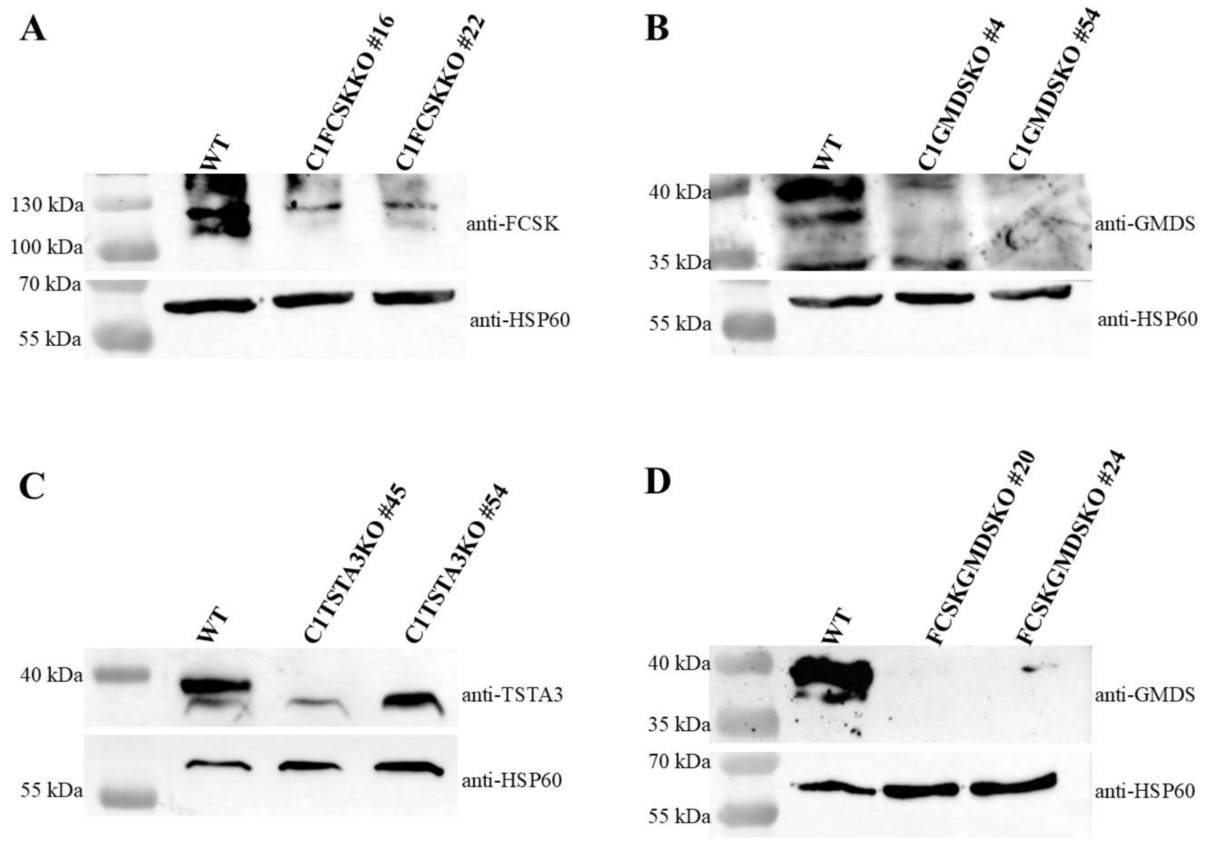

Supplementary Figure 1. Confirmation of gene inactivation in double knock-out generated in HEK293T cell line. A) Western blotting of fucokinase in the C1FCSKKO cell line. B) Cell lysates were subjected to western blotting of GMDS in the C1GMDSKO cell line. C) Western blotting of TSTA3 in the C1TSTA3KO cell line. D) Using an anti-GMDS antibody, the inactivation of the GMDS gene was verified in the FCSKGMDSKO cell line. An anti-HSP60 antibody was used for all western blots as loading controls.

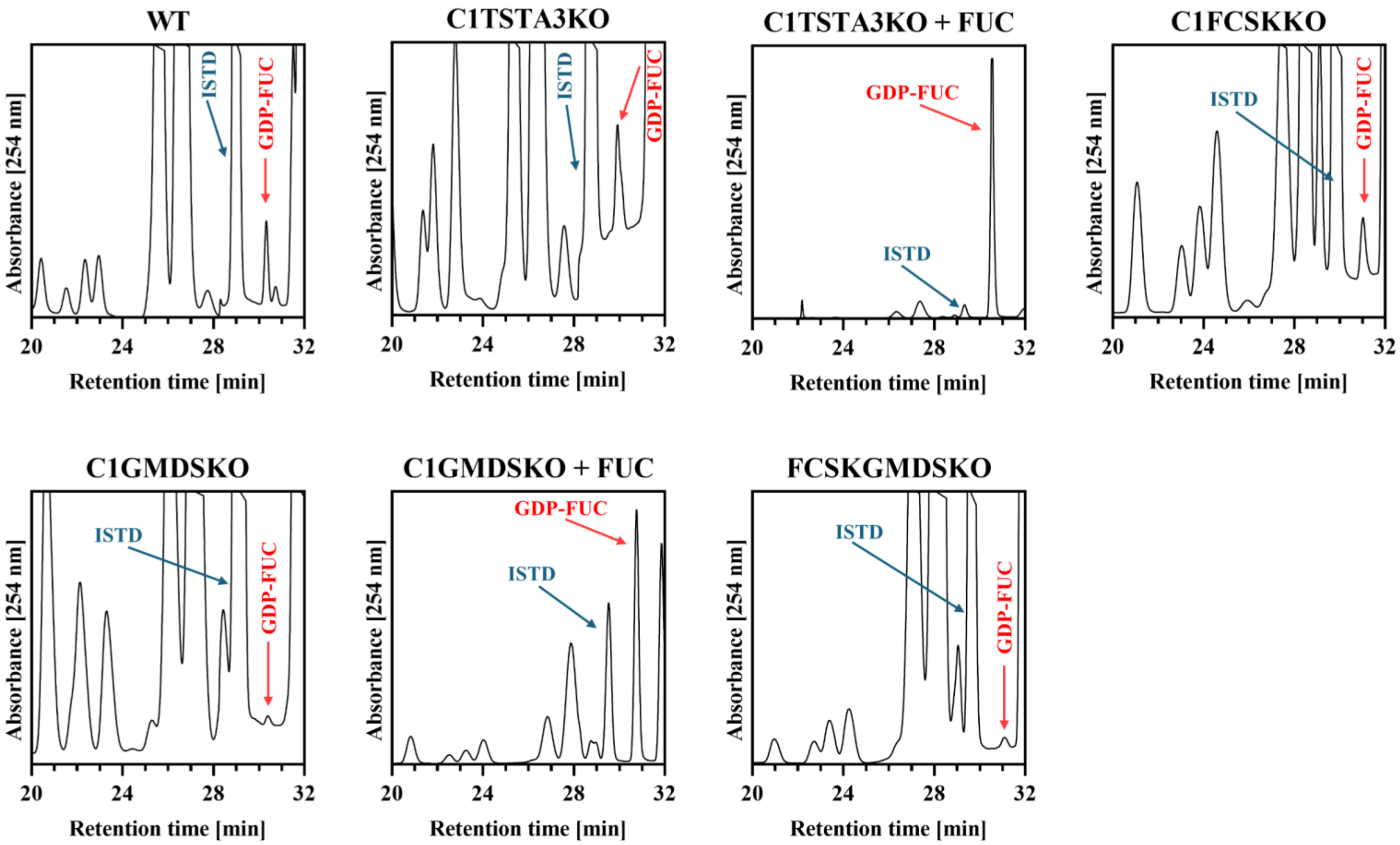

Supplementary Figure 2. An example of the separations of partially purified nucleotide sugar polls, extracted from selected cell lines. Nucleotide sugars were extracted from wild-type cells; cells deficient in C1 and TSTA3, treated with fucose (+ FUC) and untreated; cells deficient in C1 and GMDS, treated with fucose (+ FUC) and untreated, cells deficient in C1 and FCSK and cells deficient in GMDS and FCSK. Nucleotide sugars were separated using ion-pairing, reverse phase chromatography. The internal standard (GDP-glucose, ISTD) was marked in blue, and the peak corresponding to GDP-fucose was marked in red.

Supplementary Table 1. List of antibodies used in western blotting analysis

Antibody	Origin	Dilution	Manufacturer	Cat. Num.
anti-FCSK	rabbit	1:50	Novus Biologicals	NBP1-83467
anti-GMDS	rabbit	1:100	Novus Biologicals	NBP1-33424
anti-TSTA3	rabbit	1:100	Novus Biologicals	NBP1-33070
anti-SLC35C1	rabbit	1:100	Thermo Fisher Scientific	PA564146
anti-HSP60 (F-9)	mouse	1:50000	Santa Cruz Biotechnology	sc-376261
anti-mouse HRP	goat	1:10000	Promega	W4021
anti-rabbit HRP	goat	1:10000	Sigma-Aldrich	A0545
